# N-Myc and STAT interactor as a context-dependent switch in innate antiviral immunity

**DOI:** 10.3389/fimmu.2026.1864248

**Published:** 2026-06-24

**Authors:** Lin Han, Xinyao Xu, Fangfang Zhao, Luyu Mao, Yongli Guo, Mingchun Gao

**Affiliations:** 1 Department of Preventive Veterinary Medicine, College of Veterinary Medicine, Northeast Agricultural University, Harbin, China; 2 Department of Immunology, School of Basic Medical Sciences, Harbin Medical University, Harbin, Heilongjiang, China

**Keywords:** foamy virus, functional switching, human cytomegalovirus, innate antiviral immunity, interferon signaling, IRF7, N-Myc and STAT interactor

## Abstract

N-Myc and STAT interactor (NMI), initially identified as a Myc- and STAT-associated protein, is increasingly recognized as a regulatory node in innate antiviral immunity. Current evidence indicates that NMI does not exert a fixed antiviral or proviral effect. Instead, its functional output is shaped by interacting partners, signaling pathway context, subcellular localization, and stage of infection. In selected acute RNA virus models, NMI suppresses IRF7-dependent type I interferon (IFN-I) signaling. In the IAV model, this involves an NMI–IFP35 complex coupled to the TRIM21–IRF7 axis that promotes IRF7 degradation, suppresses IFN-I responses, and thereby facilitates viral replication. In foamy virus infection, by contrast, NMI directly binds the viral transactivator Tas, retains it in the cytoplasm, and suppresses viral transcription, thus acting as a host restriction factor. In human cytomegalovirus (HCMV) infection, NMI appears to function as a host pathway component targeted by viral antagonism, as the viral protein UL23 interferes with the NMI–STAT1/IFN-γ axis and dampens antiviral gene expression. In addition, extracellular NMI and IFP35 can function as damage-associated molecular patterns that amplify inflammation. Together, these findings support the view that NMI acts as a context-dependent molecular switch rather than a unidirectional effector in innate antiviral immunity.

## 1 Introduction

Antiviral innate immunity is initiated by the recognition of viral components by pattern-recognition receptors and is followed by induction of interferons, inflammatory cytokines, and interferon-stimulated genes (ISGs) (1–3). These responses must be strong enough to restrict viral replication, but also sufficiently controlled to limit tissue damage and immunopathology. In addition to type I and type III interferon pathways, IFN-γ-dependent signaling contributes to immune surveillance and downstream antiviral control (4, 5).

N-Myc and STAT interactor (NMI) has emerged as one host molecule positioned at this regulatory intersection. Initially described as a Myc- and STAT-associated protein (6), NMI was later shown to associate with IFP35, encoded by *IFI35*, and stabilize it by preventing proteasome-mediated degradation (7). Rather than acting as a classical enzymatic effector, NMI is better understood as an adaptor-like regulatory protein embedded in several antiviral and inflammatory signaling networks.

NMI does not show a uniform functional direction across viral systems. In selected acute RNA virus models, NMI suppresses type I interferon (IFN-I) responses and behaves as a proviral host factor (8–11). In foamy virus infection, however, NMI directly restricts viral transcription by targeting the viral transactivator Tas (12, 13). In HCMV infection, published evidence indicates that NMI-associated IFN-γ signaling can be targeted by the viral protein UL23 (14). In parallel, extracellular NMI and IFP35 may amplify inflammation as damage-associated molecular patterns (DAMPs) (15). These apparently contradictory observations raise a central question: why can the same host factor produce opposite outcomes in different viral contexts? Based exclusively on published studies, this Mini Review uses the term “context-dependent switch” to describe an integrative conceptual framework that links virus-specific NMI interactions, interferon signaling, subcellular localization, and inflammatory context, rather than a single experimentally resolved molecular transition.

## 2 Molecular positioning of NMI as a context-dependent regulator

NMI is best viewed as an adaptor-like regulatory protein rather than a classical enzymatic effector. Its biological function primarily depends on protein-protein interactions, which places NMI in a favorable position to integrate multiple signaling inputs. NMI is inducible by interferon and cytokine signaling, including IFN-γ and IL-2 (6, 7), and can be upregulated during viral infection (8, 11). Thus, NMI is not a constitutively inert scaffold, but an inducible immune node embedded in cytokine-responsive regulatory circuits.

A central molecular feature of NMI is its ability to form a stable complex with IFP35. NMI not only binds IFP35 but also stabilizes it by preventing proteasome-mediated degradation (7). This complex is not merely structural. In teleost models, Nmi and IFP35 mutually stabilize one another and jointly promote degradation of IRF3 and IRF7, thereby suppressing IFN-I production (16). These observations support the view that the NMI–IFP35 complex provides a functional platform for immune threshold control.

NMI also connects to distinct host signaling axes with different downstream consequences. On one side, NMI is linked to the TRIM21–IRF7 pathway and suppresses virus-triggered IFN-I production (8–10). On the other side, NMI can enhance IFN-γ signaling by limiting STAT1 SUMOylation and supporting STAT1-dependent transcriptional activity (17). TRIM21 itself is an intracellular immune regulator with diverse roles in antiviral defense and virus-host interaction (18, 19). In the NMI–IFP35 suppressive module, TRIM21 further regulates IRF7-targeted inhibition, and K63-linked ubiquitination of NMI at lysine 22 appears to contribute to activation of this complex (9, 10).

A third layer of NMI biology emerges outside the cell. Under infection- or injury-associated conditions, NMI and IFP35 can be released extracellularly and act as DAMPs, activating TLR4-NF-κB signaling and driving inflammatory cytokine production (15). This fits the broader concept that DAMPs are endogenous danger signals that activate innate immune pathways during tissue stress and sterile or infection-associated inflammation (20). Taken together, NMI links IFN-I suppression, IFN-γ enhancement, and inflammatory amplification. This multidimensional positioning provides the molecular basis for its context-dependent functional switching.

Current evidence indicates that NMI functional switching is unlikely to reflect a single linear pathway. It may instead be shaped by several mechanistic determinants: interaction-partner availability, subcellular localization, and post-translational regulation. Engagement with IFP35 and TRIM21 favors IRF7 degradation and IFN-I suppression in the IAV-related pathway (8–10), and a related NMI–IRF7–IFN-I suppressive pattern has been reported in DTMUV infection (11). Interaction with Tas supports cytoplasmic sequestration of a viral transactivator in foamy virus models (12, 13), whereas participation in the NMI–STAT1/IFN-γ axis links NMI to IFN-γ-responsive transcription (14, 17). K63-linked ubiquitination of NMI has been implicated in the NMI–IFP35 suppressive module, and NMI has also been linked to regulation of STAT1 SUMOylation, but whether these modifications directly control partner selection remains unresolved (9, 10, 17). Thus, the switch model should be viewed as a published-evidence-derived conceptual framework rather than as a structurally resolved molecular transition.

**Figure 1:**
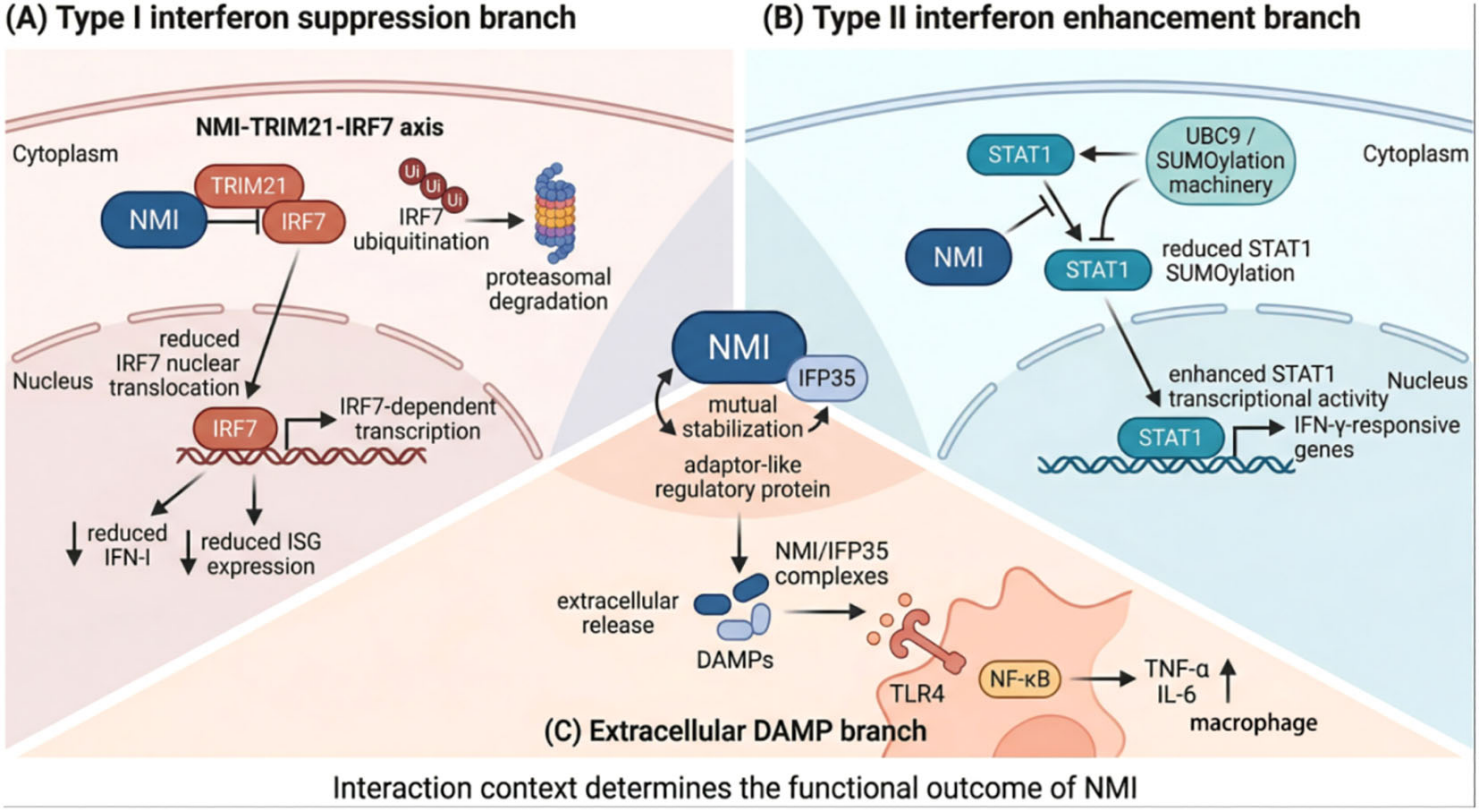
NMI as a context-dependent regulatory hub linking IFN-I suppression, IFN-γ enhancement, and extracellular DAMP signaling. **(A)** In the type I interferon suppression branch, NMI participates in the TRIM21–IRF7 regulatory axis, promotes IRF7 ubiquitination and proteasomal degradation, reduces IRF7 nuclear translocation and IRF7-dependent transcription, and thereby suppresses IFN-I production and ISG expression (8–10). **(B)** In the type II interferon enhancement branch, NMI limits STAT1 SUMOylation through the UBC9/SUMOylation machinery, enhances STAT1 transcriptional activity in the nucleus, and promotes IFN-γ-responsive gene expression (17). **(C)** In the extracellular DAMP branch, NMI and IFP35 can be released from infected or damaged cells, act as DAMPs, activate TLR4-NF-κB signaling in macrophages, and induce inflammatory mediators such as TNF-α and IL-6 (15, 20). Together, these branches illustrate that NMI functions as an adaptor-like regulatory molecule whose biological output is determined by interaction context.

For clarity, the NMI-associated mechanisms summarized in **Figure 1** can be organized into three related layers: intracellular IFN-I threshold control, IFN-γ/STAT1-associated transcriptional regulation, and extracellular DAMP-like inflammatory signaling. These layers correspond to the three branches shown in **Figure 1**.

## 3 Virus-specific functional outcomes of NMI

### 3.1 Selected acute RNA virus models: NMI as a proviral host factor

The strongest evidence for a proviral role of NMI comes from selected acute RNA virus models, particularly influenza A virus (IAV) infection (8–10). Wang et al. first showed that NMI targets IRF7 and negatively regulates virus-triggered IFN-I production (8). This was mechanistically extended by the identification of TRIM21 as a regulator of the NMI–IFP35 inhibitory complex (9). In the IAV model, Ouyang et al. demonstrated that NMI binds IRF7, recruits TRIM21, promotes IRF7 ubiquitination and degradation, and consequently suppresses IFN-I and ISG expression (10). NMI deficiency enhanced antiviral responses *in vitro* and improved survival, reduced lung viral burden, and alleviated lung pathology in infected mice (10). These findings position NMI as a host factor exploited by selected acute RNA viruses to dampen innate immunity.

A related pattern is supported in duck Tembusu virus (DTMUV) infection. Luo et al. reported that NMI is upregulated upon infection and promotes viral replication by suppressing IRF7-dependent IFN-I signaling (11). Although the full TRIM21-linked architecture remains strongest in the IAV model, the NMI–IRF7–IFN-I suppressive chain appears to extend beyond one virus species. Thus, in selected acute RNA virus models, the dominant output of NMI is to lower the IFN-I threshold and create a more permissive intracellular environment for viral replication.

### 3.2 Foamy viruses: NMI as a host restriction factor

In striking contrast, NMI behaves as a host restriction factor in foamy virus infection. Foamy viruses have a distinctive replication strategy among retroviruses, and viral transcription is strongly influenced by the viral transactivator Tas (21). Hu et al. showed that NMI directly interacts with the prototype foamy virus transactivator Tas and sequesters it in the cytoplasm, thereby reducing its ability to activate the viral long terminal repeat and internal promoter (12). In this setting, NMI does not act primarily by reshaping host interferon signaling. Instead, it acts directly on a viral regulatory protein and suppresses the transcriptional program required for efficient viral replication.

This functional inversion is mechanistically informative. It indicates that NMI does not intrinsically belong to either the “proviral” or “antiviral” category. Rather, its output depends on what it engages. If its dominant functional partner or target is a host interferon regulator such as IRF7, NMI may lower antiviral defenses. If its dominant target is a viral transactivator such as Tas, NMI can directly restrain viral replication. Earlier work on bovine foamy virus also supports this conceptual framework, as IFP35 was shown to associate with Tas and participate in interferon-mediated antiviral activity (13). Together, these data suggest that members of the NMI–IFP35-associated network can, under specific viral conditions, directly engage viral proteins and function as restriction factors.

### 3.3 HCMV: NMI as a target of viral counteraction

A third functional mode is seen in HCMV infection, where NMI becomes a target of viral antagonism. HCMV is well known for its ability to evade host immune surveillance through multiple mechanisms that interfere with antiviral responses (22). Feng et al. demonstrated that the HCMV protein UL23 interacts with human NMI and suppresses transcription of IFN-γ-stimulated genes (14). Mechanistically, UL23 interferes with the NMI–STAT1/IFN-γ axis and weakens the antiviral output of IFN-γ signaling. In this context, NMI is neither simply exploited via a preexisting host inhibitory circuit nor directly restricting a viral protein. Instead, a viral factor actively targets NMI-dependent signaling to counter host immunity.

**Figure 2:**
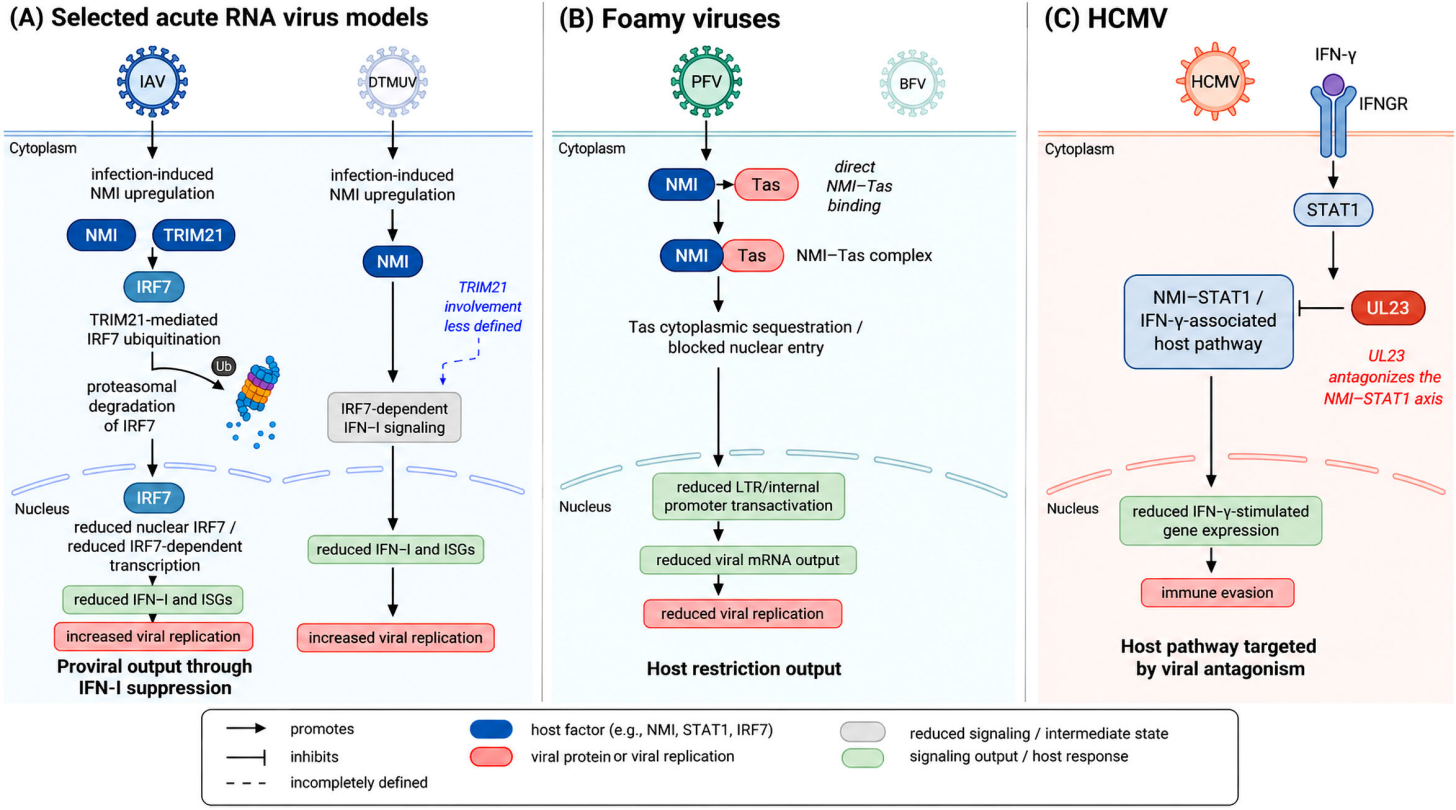
Virus-specific functional outcomes and evidence boundaries of NMI in innate antiviral immunity. **(A)** In selected acute RNA virus models, NMI can lower the IFN-I antiviral threshold. In the IAV model, published evidence supports an NMI–TRIM21–IRF7 pathway in which NMI recruits TRIM21 to IRF7, promotes IRF7 ubiquitination and proteasomal degradation, suppresses IFN-I/ISG induction, and thereby favors viral replication (8–10). In the DTMUV model, published evidence supports NMI-mediated suppression of IRF7-dependent IFN-I signaling, whereas TRIM21 involvement remains less clearly defined (11). **(B)** In foamy virus infection, NMI directly binds the viral transactivator Tas, promotes cytoplasmic sequestration of Tas, reduces LTR/internal promoter transactivation, and thereby restricts viral transcription and replication (12, 13, 21). **(C)** In HCMV infection, UL23 antagonizes an NMI–STAT1/IFN-γ-associated host pathway, reduces IFN-γ-stimulated gene expression, and promotes immune evasion (14, 22). Solid arrows indicate promotive effects, blunt-ended lines indicate inhibitory effects, and dashed lines indicate incompletely defined relationships.

This model adds a third layer to the functional landscape of NMI. In selected acute RNA virus models, NMI is largely embedded in a host negative-feedback circuit that viruses exploit. In foamy virus infection, NMI acts as a restriction factor against a viral transcriptional regulator. In HCMV infection, NMI itself becomes part of the battleground targeted by viral immune evasion mechanisms. Together, these findings indicate that the biological meaning of NMI is not fixed, but is redefined by the viral interaction context. **Figure 2** summarizes the virus-specific functional outcomes and evidence boundaries of NMI across selected acute RNA virus, foamy virus, and HCMV infection models.

## 4 Discussion

The most important implication of current studies is that NMI should not be viewed as a fixed antiviral or proviral factor, but rather as a context-dependent switch in innate antiviral immunity. Its functional output appears to be shaped by interacting partners, pathway context, subcellular localization, post-translational regulation, and infection timing. When coupled to the TRIM21–IRF7 inhibitory module, NMI tends to suppress IFN-I signaling and favor viral replication (8–11). By contrast, when directly engaging the viral protein Tas, NMI restricts viral transcription (12, 13). In IFN-γ-associated signaling, this axis may itself become a target of viral counteraction, as illustrated by UL23 in HCMV infection (14, 17, 22). These outcomes are better understood as context-specific configurations of NMI-associated signaling complexes than as mutually exclusive identities of NMI. In this review, the term “switch” denotes an integrative framework based on published evidence, not a single experimentally defined allosteric event.

Several key questions remain unresolved. Most evidence still derives from cell-based systems or individual viral models, and the structural basis of NMI interactions with IFP35, IRF7, STAT1, Tas, and UL23 remains incompletely defined. A central controversy is whether the divergent phenotypes of NMI reflect distinct molecular states of the protein itself, or whether they arise primarily from virus-dependent differences in the dominant interactome, cellular context, and stage of infection. It also remains unclear whether these partners bind compatible or mutually competitive surfaces on NMI, whether viral proteins such as Tas or UL23 displace host partners, or whether post-translational modifications alter the accessibility of interaction interfaces. In addition, intracellular signaling functions of NMI and extracellular DAMP-like activities of NMI and IFP35 are still largely discussed separately, although these processes may intersect during real infection. These gaps limit the development of a unified mechanistic framework.

One possible explanation is that the NMI–IFP35 complex functions as an immune-threshold regulator whose output depends on cell type, infection stage, and inflammatory context. In epithelial or parenchymal cells during early acute RNA virus infection, coupling of NMI to IRF7-associated inhibitory pathways may lower the IFN-I threshold and favor viral replication. In cytokine-rich environments where IFN-γ and STAT1-dependent responses dominate, NMI may support antiviral transcriptional programs. During later infection or tissue injury, extracellular NMI and IFP35 may further amplify inflammation. This interpretation helps explain why host cells may retain a bidirectional regulator: it can restrain excessive interferon activity while preserving the capacity to participate in antiviral or inflammatory responses when the dominant interaction context changes.

The IFN-I, IFN-γ, and DAMP-related branches should therefore not be viewed as fully independent modules. A more plausible model is that intracellular IFN-I suppression, IFN-γ-associated signaling, and extracellular DAMP activity form a stage-dependent continuum during infection. Early intracellular NMI may tune IRF7-dependent IFN-I output; later cytokine environments may increase the relevance of NMI–STAT1/IFN-γ signaling; and cell stress or injury may release NMI and IFP35 as extracellular danger signals. However, the temporal coordination among these branches has not yet been resolved *in vivo*.

The translational implications of NMI-associated pathways are therefore context-specific rather than universally antiviral. Inhibiting the NMI–TRIM21–IRF7 axis might theoretically restore IFN-I responses during selected early acute RNA virus infections, but the same approach could aggravate inflammatory injury if applied during later immunopathological phases. Conversely, targeting extracellular NMI or IFP35 may reduce inflammatory amplification but may not directly reduce viral replication. Broad inhibition of NMI could also be detrimental in settings where NMI supports IFN-γ/STAT1-associated host responses. Any NMI-targeted strategy would therefore need to consider viral species, infection stage, tissue compartment, cell type, and disease phenotype.

Future studies should therefore prioritize defining the structural interfaces that govern NMI interaction specificity, establishing temporally resolved *in vivo* models, and testing whether selective modulation of the NMI–TRIM21–IRF7 axis or extracellular NMI/IFP35 signaling can improve antiviral protection without aggravating immunopathology. Mechanistically, domain-mapping mutants, reciprocal co-immunoprecipitation competition assays, cross-linking mass spectrometry, and structural modeling may help determine whether NMI-binding partners occupy compatible or mutually competitive interfaces. Although such host-targeted strategies remain conceptual, the broader literature cautions that type I interferon programs can support antiviral defense while contributing to immunopathology depending on infection stage and context (23), and that ISG effector networks are functionally diverse rather than uniformly protective (24). In selected viral settings, especially where extracellular NMI/IFP35-related inflammation and interferon-associated antiviral control intersect, IFP35-centered translational evidence is emerging (15, 25).

Recent reviews of DTMUV pathogenesis and host immune regulation also highlight ongoing interest in viral–host interactions, innate immune activation, and IFN/ISG-associated antiviral mechanisms in this infection model (26). In parallel, a large-scale ISG loss-of-function atlas showed that individual ISGs can exert virus-specific antiviral or proviral effects across different viruses (27).

In summary, NMI is best understood as an interaction-dependent molecular switch whose biological meaning is determined by the protein interaction landscape in which it operates. Clarifying the structural, temporal, and *in vivo* determinants of this switching behavior will be essential for building a predictive framework of NMI function in antiviral immunity.
